# Effects of Ozonized Oil on At‐Home Tooth Bleaching: A Double‐Blind Randomized Clinical Trial

**DOI:** 10.1111/jerd.70083

**Published:** 2025-12-24

**Authors:** Murilo Guimarães Campolina, Lia Dietrich, Julia Marques Martins, Larissa Victoria Miranda Ubagai, Guilherme José Pimentel Lopes Oliveira, Ceci Nunes Carvalho, Hugo Lemes Carlo, Carlos José Soares, Gisele Rodrigues Silva

**Affiliations:** ^1^ Department of Operative Dentistry and Dental Materials, School of Dentistry Federal University of Uberlândia Uberlândia Minas Gerais Brazil; ^2^ Department of Operative Dentistry and Dental Materials, School of Dentistry, Federal University of dos Vales Do Jequitinhonha e Mucuri Minas Gerais Brazil; ^3^ Department of Periodontology & Oral Implantology Federal University of Uberlândia Uberlândia Minas Gerais Brazil; ^4^ Dental School CEUMA University Maranhão Brazil

**Keywords:** dentin desensitizing agents, dentin sensitivity, hydrogen peroxide, ozone therapy, tooth bleaching

## Abstract

**Objective:**

This double‐blind, randomized clinical trial evaluated the effect of ozonized sunflower oil on tooth sensitivity, color change, and patient perception during at‐home bleaching with 10% hydrogen peroxide.

**Clinical Considerations:**

Forty participants were randomly assigned to receive ozonized or nonozonized sunflower oil, applied in trays for 1 min after each bleaching session (30 min daily for 21 days). Sensitivity was assessed daily using visual and numerical scales. Tooth color was measured at baseline, 1 week, and 1 month after treatment using a spectrophotometer (ΔEab, ΔE00, ΔWID) and a visual shade guide (ΔSGUs). Patient perception was evaluated through Likert‐scale questionnaires.

**Conclusions:**

Overall, 72.5% of participants experienced sensitivity, with no significant difference in intensity (*p* = 0.536) or duration (*p* = 0.256) between groups. Color changes were similar (*p* > 0.05), although ΔEab and ΔE00 values decreased at 1 month (*p* = 0.010 and *p* = 0.026). Ozonized oil had an unpleasant taste (*p* = 0.002) and odor (*p* = 0.010). The use of ozonized sunflower oil did not reduce bleaching‐related sensitivity or enhance whitening effectiveness, and its poor sensory properties may limit patient acceptance.

## 1 Introduction

Tooth whitening is a globally widespread cosmetic procedure. It is a conservative and easy treatment usually performed to achieve an esthetically pleasing smile and has been the subject of numerous laboratory and clinical studies [1, 2, 3, 4]. The technique involves applying peroxide‐based whitening agents to the buccal surface of discolored teeth. The radicals produced by peroxide breakdown oxidize the organic components of the dental tissue, providing whiter teeth [5]. Tooth sensitivity is the most common adverse effect reported by patients undergoing whitening [6, 7, 8], especially when using highly concentrated hydrogen peroxide [9]. The contact of peroxide and its by‐products with the pulp causes tooth sensitivity, promoting an inflammatory response in this tissue [8]. The mean absolute risk of tooth sensitivity is approximately 51% for at‐home whitening techniques [10, 11]. The most effective approach for reducing dentin sensitivity has been the topical application of desensitizing agents, which usually work by occluding dentinal tubules or inhibiting nociceptive transmission and transduction [7, 12, 13]. While these desensitizing agents might mitigate such an adverse impact, they cannot entirely eradicate pain, despite yielding positive effects [13, 14, 15].

Acknowledged by medical sciences, ozone therapy gained prominence in dentistry as early as the 1930s [16]. Administered as a gas or dissolved in a water or oil base, ozone provides therapeutic benefits, especially anti‐inflammatory, antioxidant, and analgesic properties [17, 18]. Clinical studies have demonstrated tooth‐whitening effects through the rapid and potent oxidative degradation of discolored components and pain reduction during in‐office bleaching [18, 19]. Ozone gas has been reported to attenuate dentin sensitivity by modulating inflammatory pathways via prostaglandin and cyclooxygenase suppression [18, 19]. However, its use in managing bleaching‐induced sensitivity remains virtually unexplored.

Although gaseous ozone has demonstrated biological potential, its clinical use is limited because it requires specialized equipment, poses potential respiratory risks at high concentrations, and is chemically unstable, restricting its application primarily to in‐office protocols [17]. Ozone has also been widely investigated for its antimicrobial and disinfectant properties in various healthcare contexts, demonstrating efficacy against bacteria, fungi, and viruses under controlled conditions [20]. In dentistry, ozonized vegetable oils have emerged as a feasible alternative, maintaining ozone‐derived peroxides and ozonides for extended periods, which ensures easier handling and safe storage. When refrigerated, these oils remain stable for up to 2 years, offering a practical advantage over gaseous or aqueous forms [21]. Among the available vehicles, sunflower oil is frequently employed because of its high content of unsaturated fatty acids, favoring stable ozonide incorporation [21]. The ozonized form has shown antimicrobial, antioxidant, and wound‐healing properties and may modulate inflammatory pathways through Nrf2 activation and NF‐κB inhibition [21, 22, 23, 24]. Recent clinical studies using ozonized oils in other dental applications, such as endodontic materials, have also demonstrated favorable biological responses and biocompatibility, further supporting the translational potential of this approach [24]. Nevertheless, clinical studies assessing its performance as an adjunct in dental bleaching are still scarce.

Considering its physicochemical stability and reported anti‐inflammatory potential, ozonized sunflower oil represents a promising but understudied adjunct for at‐home bleaching procedures, particularly for managing peroxide‐related sensitivity. However, the available evidence regarding its clinical performance remains limited, and its influence on bleaching efficacy and patient‐reported outcomes is unknown. Therefore, evaluating ozonized sunflower oil under controlled clinical conditions is relevant, given that tooth sensitivity continues to be the most frequent and limiting adverse effect associated with at‐home bleaching [10, 11, 22].

The objective of this randomized clinical trial was to evaluate the effects of experimental ozonized sunflower oil on bleaching‐related tooth sensitivity, color change, and patient perception during at‐home bleaching with 10% hydrogen peroxide. The comparison between ozonized and nonozonized sunflower oil was based on their clinical feasibility, stability, and potential anti‐inflammatory properties.

The hypotheses of this study are that significant differences will be observed between ozonized sunflower oil and nonozonized sunflower oil regarding (1) the absolute risk and intensity of bleaching‐induced tooth sensitivity, (2) the effectiveness of bleaching in terms of tooth color change (ΔEab, ΔE00, ΔWID, and shade guide units), and (3) patient perception of treatment, including taste, odor, handling, and satisfaction.

## 2 Materials and Methods

### 2.1 Ethics Approval and Protocol Registration

This trial was approved by the Research Ethics Committee on Human Subjects of the Federal University of Uberlândia, Uberlândia, MG, Brazil (CAAE: 68506423.5.0000.5152) and registered in the Clinical Trials Registry under RBR‐6V7M9ZK. The research started after the analysis, adequacy, and acceptance of volunteers. The selected volunteers were informed about the research protocol, and those who agreed to participate signed an informed consent form.

### 2.2 Trial Design and Location

This study is a randomized, parallel, and double‐blind controlled trial. The allocation ratio was equal between groups and designed according to the guidelines of the Consolidated Standards of Reporting Trials (CONSORT) [25]. It was conducted at the School of Dentistry of the Federal University of Uberlândia (FOUFU), Uberlândia, MG, Brazil from September 2023 to April 2024.

### 2.3 Sample Size Estimation, Recruitment, Eligibility Criteria, and Allocation

Considering tooth sensitivity as the primary outcome in this study, with an approximate 60% absolute risk [26] in a clinically relevant parallel design, the sample size was calculated for a superiority test with a binary outcome, with an 80% power and a 5% significance level. Thus, a minimal sample size of 20 participants per group was required to detect a decrease in the primary outcome measure from 60% to 30% using the ozonized oil.

Recruitment was conducted through social media. Volunteers were informed of the study objectives and signed an informed consent form before enrollment. Patients were selected at the Dental Clinic of the School of Dentistry at the Federal University of Uberlândia. Initially, a detailed anamnesis was performed for each case, followed by professional prophylaxis using a rubber cup with pumice paste (SS White, Rio de Janeiro, RJ, Brazil) and water to remove extrinsic stains and facilitate the clinical examination. Two examiners performed this examination to verify inclusion and exclusion criteria.

**FIGURE 1:**
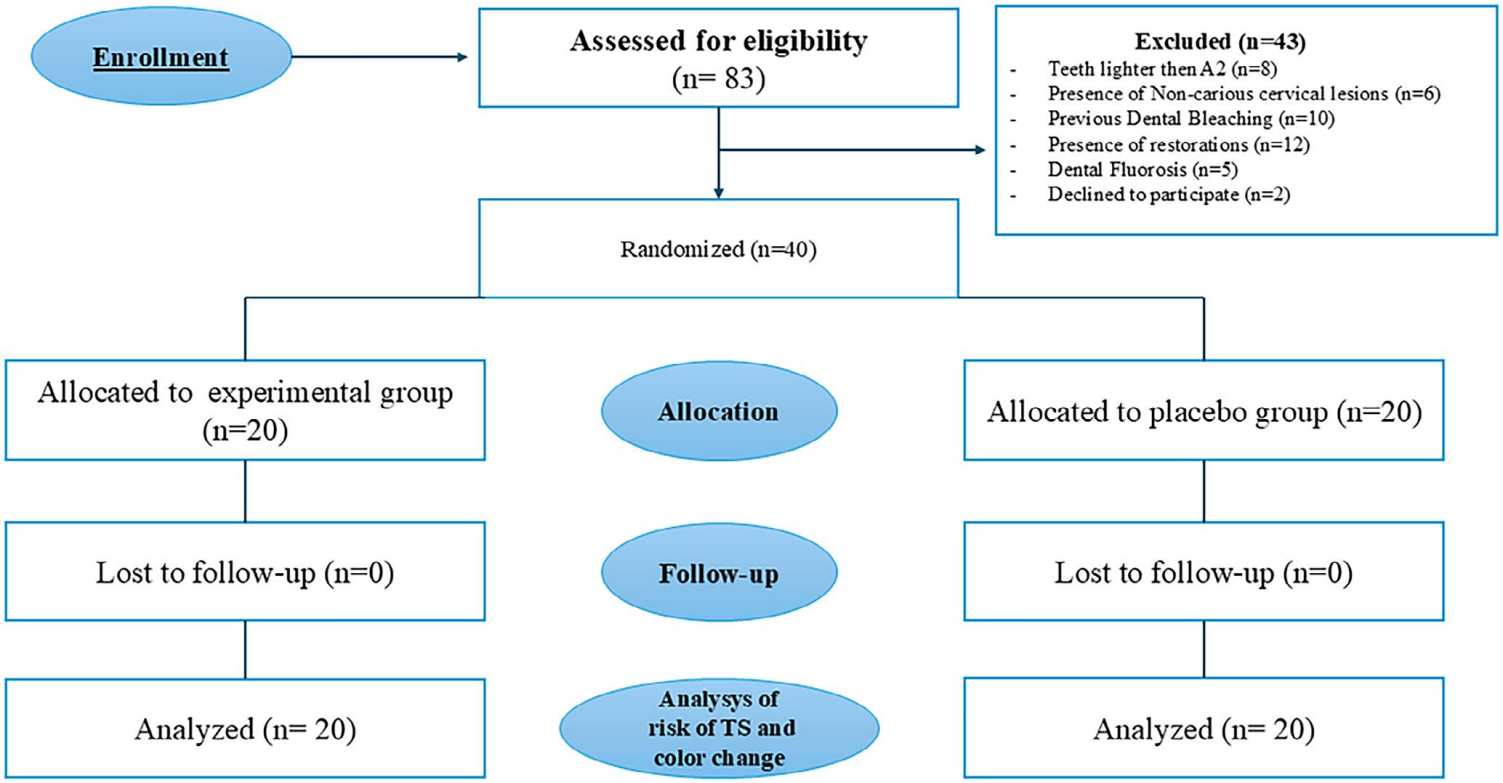
Schematic depiction delineating study progression, including participant recruitment and allocation into the respective groups.

As in Figure 1, 83 patients were evaluated. The study included 40 patients between 18 and 40 years old with good oral and general health and anterior maxillary teeth devoid of restorations and carious lesions. Canine tooth color was established as A2 or darker, according to the Vita Classic shade guide (Vita Zahnfabrik, Bad Säckingen, Germany) for inclusion in the study.

Forty‐three patients were excluded (Figure 1). The exclusion criteria comprised patients with severe discoloration (e.g., stains caused by tetracycline), enamel hypoplasia, gingival recession, dentin exposure, visible enamel cracks, pulpitis, or endodontic treatment in any of the six upper anterior teeth. The study also excluded participants with a history of whitening procedures and tooth sensitivity, continuously taking anti‐inflammatory or analgesic drugs, smokers, engaged in parafunctional habits, undergoing treatment with fixed or removable orthodontic appliances, pregnant, or lactating.

The patients were randomly allocated into two groups (*n* = 20) according to the oil. All participants underwent a 21‐day regimen of at‐home bleaching with a daily 30‐min gel application (10% hydrogen peroxide gel, *White Class*, FGM, Joinville, Brazil). After applying the whitening agent, the oils were set into individual trays for 1 min. The experimental group received daily applications of ozonized oil, while the control group received sunflower oil as their respective allocations. Ten percent of hydrogen peroxide was applied according to the manufacturer's instructions. Table 1 describes the products used in the study, their compositions, and application methods.

**TABLE 1:** Manufacturer, composition, and application method of the primary products analyzed in this study.

Manufacturer	Composition	Application method
White class 10% (FGM, Joinville, SC, Brazil)	10% hydrogen peroxide, neutralizer, potassium nitrate, sodium fluoride, calcium gluconate, stabilizer, humectant, and deionized water	Daily 30‐min gel application for 21 days using individual trays
Ozonized sunflower oil (Philozon, Balneário Camboriú, SC, Brazil)	*Helianthus annuus* (sunflower) seed oil and ozone (629 meq/kg peroxide concentration)	Daily 1‐min application after the bleaching gel
Sunflower oil (Liza, São Paulo, Brazil)	*Helianthus annuus* (sunflower) seed oil	Daily 1‐min application after the bleaching gel

### 2.4 Randomization and Blinding

Clinical files designed for the study originated from personal patient information, data on the characteristics of involved teeth, and patient signatures. All documents handled during the visit were coded with an Arabic number to protect the anonymity of the names of volunteers.

Blocked randomization (block size of 2) ensured equal‐sized groups with equal allocation ratios at www.sealedenvelope.com. A person unrelated to the intervention or assessment (L.V.M.U.) computer‐generated a random list. The allocation sequence was inserted into sealed envelopes numbered from 1 to 40 and opened by the operator (M.G.C.) only at the time of intervention. Patients were numbered according to the allocation sequence.

It was a double‐blind study in which evaluators (J.M.M. and H.L.C.) and patients were unaware of the material applied along with whitening. The operators could not be blinded because ozone has a typical pungent odor, and they had used it in other studies.

Both ozonized and nonozonized sunflower oils were dispensed in identical opaque 5‐mL syringes, coded by a researcher not involved in the intervention. The syringes were indistinguishable in appearance and delivered directly to the patients according to their allocation group. This ensured that patients and evaluators (JMM and HLC) remained blinded to the intervention. Operators could not be blinded, as they had previously handled ozonized oil in other studies and might recognize its characteristic odor. Although ozonized oil presents a distinctive odor, patients were not informed about this characteristic to avoid expectation bias, and both groups received identical application instructions. This limitation was acknowledged and considered in the interpretation of patient perception outcomes.

### 2.5 Intervention

During patient selection, an initial step involved professional prophylaxis using a rubber cup with pumice paste (SS White, Rio de Janeiro, RJ, Brazil) and water to eliminate extrinsic stains and facilitate subsequent clinical examinations. The patients enrolled in the study underwent upper and lower dental arch impressions using alginate material (Dentsply Sirona, Milford, DE, United States).

After disinfection with 70% alcohol spray and water, plaster models were generated (Asfer Indústria Química Ltda., São Caetano do Sul, SP, Brazil). These plaster models helped craft customized top and bottom 2 mm acetate trays for each patient (Whiteness Plates for Molders, FGM, Joinville, SC, Brazil) using a vacuum plasticizer (Plastivac P7, BioArt, São Carlos, SP, Brazil). Excess material on labial and lingual surfaces was removed, providing a 1 mm distance from the gingival margin. Subsequently, the trays underwent fitting tests in patients and were adjusted accordingly.

Both groups used a hydrogen peroxide‐based whitening agent at 10% concentration (White Class, FGM, Joinville, SC, Brazil). The volunteers received the tray, the whitening gel tube, and a 5 mL syringe containing the oil from their allocation groups and the following instructions:

1. Press the syringe plunger and apply the whitening gel to the tray indentations. Typically, a small drop per tooth is adequate for coverage.
2. Fit the tray onto the teeth and lightly press to ensure the gel surrounds them
3. Apply the gel for 21 days for 30 min daily.
4. Rinse the tray before storage and before each use. Store the gel in a ventilated place, preferably in a refrigerator. Avoid prolonged syringe exposure to light. Discard empty syringes.
5. Use the individual tray for the daily oil application right after bleaching. Pour one oil drop on each tooth of the tray and place it over the bleached teeth for 1 min.

After use, patients were instructed to wash the trays with running water and neutral soap, dry them before storage, and brush their teeth to ensure adequate oral hygiene and removal of residual oil. Both ozonized and nonozonized sunflower oils presented a liquid oily consistency, with no thickening agents in their formulation.

The 21‐day (3‐week) at‐home bleaching protocol was adopted based on previous randomized clinical trials evaluating low‐concentration hydrogen peroxide, reporting that daily application periods in at‐home bleaching ranged from 6 to 28 days, which ensures adequate cumulative exposure and stable color outcomes [10]. Accordingly, extending the treatment to 21 days allowed the whitening effect to reach its plateau while maintaining patient safety regarding tooth sensitivity.

### 2.6 Color Change

Two calibrated evaluators (J.M.M. and H.L.C.) with superior color discrimination competency according to the ISO/TR 28642 and an inter‐examiner agreement level of at least 85% (Kappa statistic) assessed the color before bleaching (baseline), 1 week after bleaching (1‐week), and 1 month after bleaching (1‐month). The color evaluation immediately after concluding the bleaching process was purposely excluded to prevent the potential dehydration and demineralization effects on color readings. The value‐centric shade guide Vita Classical (Vita Zahnfabrik) and the Vita Easyshade spectrophotometer (Vita Zahnfabrik) evaluated tooth color. The 16 Vita Classical shade guide tabs were placed in descending order from highest (B1) to lowest (C4) for a subjective rating. We used the middle portion of the right upper canine for tooth matching. Color changes were tracked from the active phase to recall times using ΔSGUs, concentrating on lighter hues on the value‐oriented shade tab list. When assessors disagreed, a consensus had to be established before discharging the patient.

A cast of the upper arch was created with condensation silicone (gray color, Maquira Dental Group, Maringá, PR, Brazil) for use with the Vita Easyshade spectrophotometer (Vita Zahnfabrik) to standardize the color measurement area throughout the experiment. A 6‐mm‐diameter perforation was made in the silicone index corresponding to the buccal surface of the upper right canine to allow standardized positioning of the spectrophotometer tip (Easyshade Compact Advance 4.0, Vita Zahnfabrik, Bad Säckingen, Germany).

The L* (brightness), a* (hue on the red–green axis), and b* (hue on the blue–yellow axis) values in the canine were measured in triplicate, and the measurement mean was recorded [27, 28, 29]. The color change result (difference between baseline 1‐week and baseline 1‐month) was calculated with three methods [27, 28, 29]: CIELab:ΔEab = [(ΔL*)2 + (Δa*)2 + (Δb*)2] × 1/2; CIEDE2000 formula: ΔE00 = [(Δ*L*/kLSL)2 + (Δ*C*/kCSC)2 + (Δ*H*/kHSH)2 + RT (Δ*C* × Δ*H*/SC × SH)], and the whiteness index, ΔWID = (0.511L*) − (2.3424a*) − (1.100b*). As for color change assessment, the perceptibility thresholds were established at ΔEab = 1.2 and ΔE00 = 0.8, while acceptability thresholds were set at ΔEab = 2.7 and ΔE00 = 1.8 [27, 28, 29].

### 2.7 Tooth Sensitivity

Patient‐reported tooth sensitivity was recorded daily during the 21 days of the bleaching protocol using the universal pain assessment tool, considering a 0–10 visual analog scale (VAS) and a five‐point numerical rating scale (NRS) [30, 31].

The VAS comprises a 10 cm line with endpoints labeled as 0–10. Zero means no sensitivity, and 10 means severe tooth sensitivity. Participants were asked to indicate tooth sensitivity intensity by drawing a vertical line along the horizontal VAS. Subsequently, the distance in millimeters from point zero was measured with a millimeter ruler to indicate the level of recorded tooth sensitivity.

Tooth sensitivity was also scored by the five‐point numerical rating scale (NRS: 0 = *none*, 1 = *mild*, 2 = *moderate*, 3 = *considerable*, and 4 = *severe*).

The statistical analysis considered the highest score (NRS) or numeric value (VAS) recorded during all assessments. The NRS defined the presence (score different from zero) or absence of tooth sensitivity in all assessments. This binary outcome defined tooth sensitivity risk, representing the proportion of participants reporting tooth sensitivity at least once during the treatment and the number of days they experienced pain. Meanwhile, the VAS determined tooth sensitivity intensity based on the highest daily‐recorded numbers from each patient according to allocation groups.

### 2.8 Patient Perception

After the 21‐day bleaching regimen and oil applications, the participants completed a questionnaire to evaluate their experience. This self‐administered questionnaire comprised five statements. Given the characteristic odor and taste of ozone oil, two statements inquired about the subjective sensations experienced by patients, another statement addressed the ease of handling the materials, and the final two statements sought feedback on patient satisfaction with whitening outcomes and gum irritation experienced during treatment.

The patients used a Likert scale to score each statement: (1) *completely disagree*, (2) *partially disagree*, (3) *no opinion*, (4) *partially agree*, and (5) *completely agree* [32].

### 2.9 Data Analysis

The statistician (G.J.P.L.O.) involved in the study was blinded to group allocations. The data collected from the analyses regarding the experimental ozonized oil and the placebo were compiled. Jamovi software provided the statistical analyses, with all tests performed at a 5% significance level. The assumptions of normal distribution (Shapiro–Wilk test) and equal variance (Levene's test) of the continuous data were inspected.

The Mann–Whitney test compared tooth sensitivity intensity in the NRS scale and patient perception, and the t‐test compared VAS data and the number of days experiencing pain. Fisher's test compared tooth sensitivity risk data. Color data (ΔEab, ΔE00, ΔWID, and ΔSGU) were analyzed for treatment comparison (Sunflower oil vs. Ozonized Sunflower Oil) across different time points (1‐week and 1‐month) using repeated measures ANOVA (RM ANOVA).

## 3 Results

### 3.1 Characteristics of the Included Participants and Study Adherence

Eighty‐three individuals were initially assessed for eligibility according to predefined inclusion and exclusion criteria (Figure 1). The baseline tooth color of the canine in ΔSGUs was similar between groups (ozonized oil: 10.7 ± 1.0; placebo: 10.6 ± 1.1). The patients were predominantly women (67.5%). Most participants were young adults, with an average age of 22.3 ± 1.7. The 40 participants diligently attended the scheduled follow‐up visits during the study.

### 3.2 Tooth Sensitivity Risk and Intensity

**FIGURE 2:**
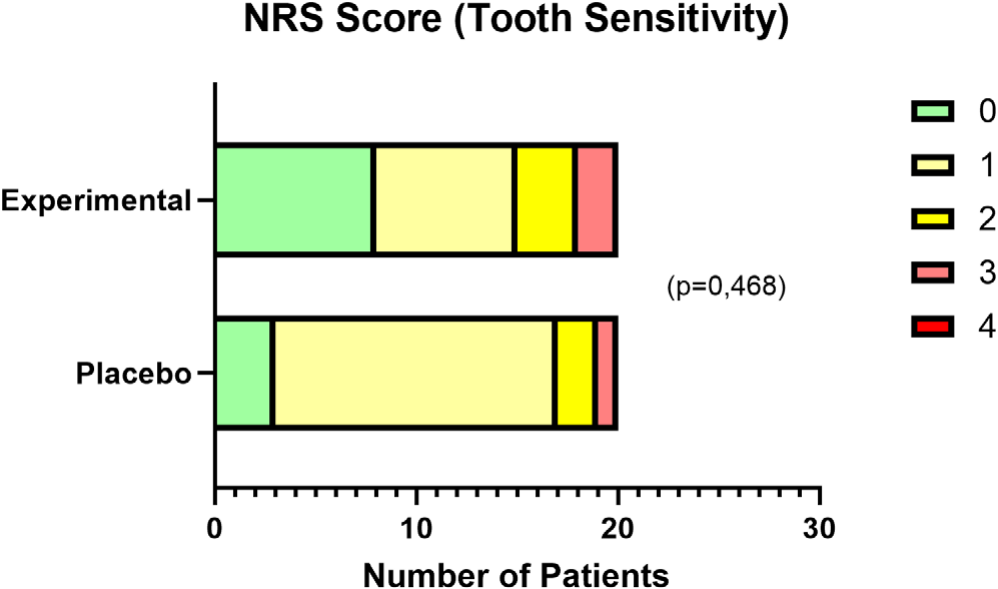
NRS scores for tooth sensitivity, indicating its presence and intensity as reported by patients: 0 = *none*, 1 = *mild*, 2 = *moderate*, 3 = *considerable*, and 4 = *severe sensitivity*. Fisher's test compared absolute and relative risks and tooth sensitivity intensity. Regardless of the treatment, no patient reported severe tooth sensitivity (4).

Regardless of the treatment, 72.5% of participants experienced tooth sensitivity during at‐home bleaching without reporting severe pain scores (NRS = 4) (Table 2; Figure 2). The statistical analysis revealed no significant differences between the experimental ozonized oil and the placebo group regarding pain intensity (*p* = 0.468) and the number of days experiencing tooth sensitivity (*p* = 0.256) reported by patients throughout the 21‐day bleaching period (Table 3; Figure 2).

**TABLE 2:** Tooth sensitivity risk at any assessment time, according to the group.

Group	No. of patients with any TS/Total no.	Absolute risk (%)	Relative risk^a^ (95% CI)	*p*
Sunflower oil	17/20	85%	1.4 (0.9–2.1)	0.2
Ozonized sunflower oil	12/20	60%		

^a^ Relative risk calculated with Fisher's exact test. TS—tooth sensitivity. CI—confidence interval.

**TABLE 3:** Tooth sensitivity intensity and the number of days experiencing pain for both groups in means and standard deviations according to the VAS.

Pain scales	Groups		*p*
	Sunflower oil	Ozonized sunflower oil	
VAS 0–10	1.6 ± 1.3	1.3 ± 1.7	0.54
Number of days experiencing pain	3.7 ± 3.3	2.5 ± 3.2	0.26

### 3.3 Color Evaluation

All groups exhibited significant color change. Four color evaluation tools assessed color change: ΔEab, ΔE00, ΔWID, and ΔSGU (Table 4). The analysis revealed no significant differences in color change between the groups (*p* > 0.05). However, ΔEab and ΔE00 values at 1 month were lower than at 1 week (*p* = 0.010 and *p* = 0.026, respectively) (Table 4). Although the color change was significant between the evaluation times (1‐week and 1‐month) when using the Vita Easyshade spectrophotometer (ΔEab, ΔE00), the operators did not identify visual differences using the Vita Classical shade guide (ΔSGU) at 1 week and 1 month (*p* = 0.967).

**TABLE 4:** Means and standard deviations of ΔEab, ΔE00, ΔWID, and ΔSGU from baseline to 1 week and 1 month after bleaching.

Color evaluation tool	Groups (1‐week)		*p* ^a^	Groups (1‐month)		*p* ^b^
	Sunflower oil	Ozonized sunflower oil		Sunflower oil	Ozonized sunflower oil	
ΔEab	8.0 ± 3.3 Aa	9.2 ± 3.1 Aa	0.22	7.6 ± 3.3 Ba	8.9 ± 3.0 Ba	0.01
ΔE00	5.4 ± 2.4 Aa	5.4 ± 1.8 Aa	0.92	5.2 ± 2.4 Ba	5.3 ± 1.8 Ba	0.03
ΔWID	11.4 ± 6.4 Aa	14.0 ± 6.2 Aa	0.15	10.9 ± 6.2 Ba	14.1 ± 6.2 Ba	0.60
ΔSGU classical	7.6 ± 1.8 Aa	7.6 ± 2.0 Aa	0.97	7.5 ± 1.8 Aa	7.6 ± 2.0 Aa	0.32

*Note*: Different lowercase letters (a, b) indicate significant differences between groups at the same time point. Different uppercase letters (A, B) indicate significant differences between evaluation times within the same group.

^a^ Comparisons between groups at each assessment time.

^b^ Comparisons between evaluation times (1‐week or 1‐month).

### 3.4 Patient Perception Assessment

Patients used a Likert scale to evaluate their perception during treatment, with ratings from 1 (*completely disagree*) to 5 (*completely agree*) (Table 5). Compared to the placebo, patients' perception of the ozonized oil was negative regarding taste (*p* = 0.005), odor (*p* = 0.013), and applicability (*p* = 0.048). Patients expressed overall satisfaction with the whitening outcomes achieved by the protocol. Furthermore, the use of ozonized and nonozonized sunflower oil experienced similar levels of gum irritation during treatment (Table 5).

**TABLE 5:** Medians (interquartile ranges) of patient perceptions regarding the oils and bleaching outcomes.

Statements	Sunflower oil	Ozonized sunflower oil	*p*
The oil flavor was pleasant	4 (3.8–5)	2 (1–3)	0.002
The oil smell was pleasant	4 (3–4)	2 (1.8–3)	0.01
Oil application was easy	5 (4.8–5)	4 (4–5)	0.04
The whitening outcome was satisfactory	5 (4–5)	5 (4–5)	0.6
Gum irritation occurred during whitening	4 (2–4)	2.5 (1–4)	0.2

*Note*: Scoring system: 1 = *completely disagree*; 2 = *partially disagree*; 3 = *no opinion*; 4 = *partially agree*; 5 = *completely agree*.

## 4 Discussion

The present study showed that ozonized sunflower oil did not reduce the risk or intensity of bleaching‐induced tooth sensitivity when compared with nonozonized sunflower oil. Based on these results, the first research hypothesis was rejected. Ozone has been reported to promote analgesic and anti‐inflammatory effects by reducing prostaglandin synthesis and cyclooxygenase activity, in addition to stimulating local antioxidant responses and tissue repair [16, 18, 21]. However, such effects appear to depend on the ozone form, exposure time, and concentration used [17, 19]. In this study, the 1‐min topical application of ozonized oil may have been insufficient to allow diffusion of ozonides through enamel and dentin or to trigger the pulpal anti‐inflammatory response described in previous reports [16, 18]. Moreover, clinical and laboratory investigations have demonstrated that ozone's biological performance differs considerably depending on whether it is applied as a gas, aqueous solution, or lipid‐based compound [17, 19, 23]. The brief and superficial interaction of the oil with dental tissues may therefore explain the absence of measurable desensitizing effects under the tested conditions.

Although conventional desensitizing agents, such as potassium nitrate and glutaraldehyde‐based products, are commonly used to alleviate bleaching‐induced sensitivity, their clinical effectiveness remains partial, with a significant proportion of patients still experiencing discomfort during treatment [13].– [15] This limitation justifies the search for alternative agents capable of modulating pulpal inflammation rather than merely providing neural or tubular blockage. In this context, ozonized sunflower oil was hypothesized to act through a distinct biological mechanism based on anti‐inflammatory modulation and tissue protection [15, 21, 24]. Its chemical stability, particularly under refrigeration, and ease of domiciliary application without the need for specialized equipment were considered additional advantages over traditional desensitizers [21, 23]. These attributes could theoretically expand its clinical applicability, particularly for at‐home bleaching protocols. However, under the tested conditions, these theoretical benefits did not translate into measurable clinical improvements in bleaching‐induced tooth sensitivity.

The second hypothesis, that ozonized sunflower oil would influence bleaching efficacy, was also rejected. The ozonized oil did not affect color change, neither enhancing nor reducing the whitening effect. Tooth bleaching efficacy is mainly attributed to the oxidative action of free radicals generated during hydrogen peroxide breakdown, which oxidize organic pigments within the enamel and dentin, increasing light reflection and resulting in a lighter tooth shade [5, 14, 17, 18]. Specific ozone concentrations suitable for at‐home bleaching protocols remain poorly defined, and the peroxide content in the oil tested in this study (629 meq/kg) was likely insufficient to produce additional oxidative or synergistic effects. Similar outcomes have been reported in previous studies, which found that combining ozone with hydrogen peroxide did not significantly enhance bleaching efficacy [17, 18, 19].

A mild rebound effect was observed 1 month after bleaching, consistent with the partial color regression typically attributed to enamel rehydration [14]. Although statistically significant differences were detected in ΔEab and ΔE00 between the 1‐week and 1‐month assessments, these variations remained below the established perceptibility and acceptability thresholds (ΔEab = 1.2/2.7; ΔE00 = 0.8/1.8) [28, 29]. In addition, no visual differences were perceived according to the VITA Classical shade guide (ΔSGU), indicating that the rebound effect observed instrumentally had no clinical relevance. Therefore, despite minor numerical differences, the color stability observed in both groups confirms that ozonized sunflower oil did not interfere with the whitening outcome.

The third hypothesis, that ozonized sunflower oil would positively influence patient perception, was rejected. Patient perception was a vital aspect evaluated in this research, as it is often disregarded in randomized controlled trials on tooth bleaching and adjunctive therapies, which usually focus on objective parameters such as tooth sensitivity or shade change [6, 10, 14, 22]. Subjective responses, however, are crucial for assessing the clinical viability of novel agents, particularly in at‐home protocols that depend on patient adherence. After completing the 21‐day treatment, participants rated their experiences through a structured questionnaire. Although most patients reported satisfaction with the bleaching results regardless of the method, those using ozonized oil expressed significantly lower satisfaction due to its pungent odor and unpleasant taste, which compromised comfort and ease of use. The distinct odor of ozone, chemically associated with its oxidative reactivity, has long been described as a limiting factor for patient compliance in ozone‐based therapies [21, 23, 32]. Despite these drawbacks, no participants reported adverse oral reactions or gingival irritation, suggesting that the main limitation of the ozonized oil lies in its sensory tolerability rather than biological compatibility. This finding reinforces the need for improved formulations or masking agents to enhance patient acceptance of ozonized products in home‐based bleaching regimens.

Several limitations of the present study should be acknowledged. First, the 1 min application time of the ozonized sunflower oil may have been insufficient to produce significant pulpal or dentinal effects. Second, the concentration of peroxide within the ozonized oil was standardized according to manufacturer information, but variations in ozonation degree or peroxide index could influence the biological performance of the compound. Third, the sample size, although consistent with previous clinical trials on bleaching sensitivity, may limit the detection of subtle differences in patient‐reported outcomes. Finally, patient perception was assessed through self‐reported questionnaires, which, while essential for understanding subjective experiences, may introduce response bias.

Future investigations should explore longer contact times, different ozonation levels, and alternative delivery systems, such as mucoadhesive or slow‐release formulations, to enhance the interaction of ozonides with dental tissues. Comparative studies involving other vegetable oils or ozonated matrices could also help elucidate the influence of the lipid vehicle on clinical outcomes. Additionally, integrating biochemical and histological analyses would provide valuable insights into pulpal inflammatory markers and tissue‐level responses. Collectively, these efforts could help establish more effective and patient‐tolerant ozonized products for at‐home dental bleaching applications.

## 5 Conclusion

Within the limitations of this current study, it was concluded that (1) ozonized sunflower oil did not reduce bleaching‐induced tooth sensitivity, (2) it did not affect the bleaching outcome; however, (3) patients reported a negative perception of its use due to unpleasant taste and odor.

## Author Contributions

**Murilo Guimarães Campolina** and **Lia Dietrich:** investigation, methodology, formal analysis, and writing – original draft. **Julia Marques Martins**, **Hugo Lemes Carlo**, and **Larissa Victoria Miranda Ubagai:** investigation, methodology, formal analysis, and writing – review and editing. **Guilherme José Pimentel Lopes Oliveira:** data curation, formal analysis, and resources. **Ceci Nunes Carvalho:** supervision, writing – review and editing. **Carlos José Soares:** conceptualization, funding acquisition, and writing – review and editing. **Gisele Rodrigues Silva:** conceptualization, funding acquisition, project administration, resources, and writing – review and editing.

## Funding

This study was partially funded by the Coordinator for the Improvement of Higher Education Personnel—Brazil (CAPES) (Finance Code 001), with additional support from the National Council for Scientific and Technological Development—Brazil (CNPq); National Institutes of Science and Technology (INCT)—Oral Health and Dentistry (406840/2022‐9), and the Minas Gerais Research Foundation (FAPEMIG; APQ‐02621‐21 and REDE MINEIRA EM SAÚDE ORAL E ODONTOLOGIA; RED‐00204‐23).

## Ethics Statement

This clinical investigation was approved by the Research Ethics Committee on Human Subjects of the Federal University of Uberlândia, Uberlândia, MG, Brazil (CAAE: 68506423.5.0000.5152) and registered in the Clinical Trials Registry under RBR‐6V7M9ZK.

## Consent

All participants provided informed consent before their inclusion in the study. Any identifying details that could reveal the subjects' identities were excluded. Informed consent was secured from each individual participant involved in the study.

## Conflicts of Interest

The authors declare no conflicts of interest.

## Data Availability

The data that support the findings of this study are available from the corresponding author upon reasonable request.
